# Severe lower urinary tract symptoms due to anteriorly located midline prostatic cyst arising from the bladder neck in a young male: case report: Sintomas graves do trato urinário inferior em decorrência de cisto anteriormente localizado na linha mediana da próstata proveniente do colo vesical em um jovem do sexo masculino: relato de caso

**DOI:** 10.1590/1516-3180.2016.0056280516

**Published:** 2016-09-26

**Authors:** Ali Gürağaç, Zafer Demirer, Bilal Fırat Alp, Emin Aydur

**Affiliations:** I MD. Specialist, Department of Urology, Tatvan Military Hospital, Bitlis, Turkey.; II MD. Specialist, Department of Urology, Eskişehir Military Hospital, Eskişehir, Turkey.; III MD. Associate Professor, Department of Urology, School of Medicine, Gulhane Military Medical Academy, Ankara, Turkey.

**Keywords:** Cysts, Prostate, Prostatic diseases, Urinary bladder, Urinary bladder neck obstruction, Cistos, Próstata, Doenças prostáticas, Bexiga urinária, Obstrução do colo da bexiga urinária

## Abstract

**CONTEXT:**

Prostatic cysts are uncommon. These cysts are usually asymptomatic and are diagnosed incidentally during ultrasonographic examination. On rare occasions, they may cause drastic symptoms.

**CASE REPORT:**

We report on a case of severely symptomatic anteriorly located prostatic cyst arising from the bladder neck in a 30-year-old man presenting with lower urinary tract symptoms, without clinical evidence of benign prostatic hyperplasia. Transrectal ultrasonography (TRUS), computed tomography (CT) and cystourethroscopy demonstrated a projecting prostatic cyst that occupied the bladder neck at the precise twelve o’clock position. It was acting as a ball-valve, such that it obstructed the bladder outlet. Transurethral unroofing of the cyst was performed and the patient’s obstructive symptoms were successfully resolved. Histopathological examination indicated a retention cyst.

**CONCLUSIONS:**

It should be borne in mind that midline prostate cysts can be a reason for bladder outlet obstruction in a young male. Such patients may have tremendous improvement in symptoms through transurethral unroofing of the cyst wall.

## RESUMO

**CONTEXTO:**

Cistos prostáticos são incomuns. Esses cistos são geralmente assintomáticos e são diagnosticados incidentalmente durante o exame ultrassonográfico. Raramente podem causar sintomas importantes.

**RELATO DE CASO:**

Relatamos um caso sintomático de grave cisto prostático de localização anterior, originário do colo da bexiga de um homem de 30 anos de idade, que apresentou sintomas do trato urinário inferior, sem evidência clínica de hiperplasia prostática benigna. Ultrassonografia transretal (TRUS), tomografia computadorizada (CT) e cistouretroscopia demonstraram um cisto prostático saliente que ocupou o colo da bexiga na posição exata de 12 horas. O cisto estava agindo como uma válvula de esfera, obstruindo a saída da bexiga. Retirada da cobertura do cisto foi realizada por via transuretral e os sintomas obstrutivos do paciente foram resolvidos com sucesso. O exame histopatológico indicou um cisto de retenção.

**CONCLUSÕES:**

Deve ser lembrado que a linha média do cisto de próstata pode ser motivo de obstrução da saída da bexiga em um jovem do sexo masculino. Esses pacientes podem ter notável melhoria nos sintomas com retirada da cobertura por via transuretral da parede do cisto.

## INTRODUCTION

Over recent years, the widespread availability of transrectal ultrasound (TRUS), computed tomography (CT) and magnetic resonance imaging (MRI) has led to increased frequency of diagnoses of incidental prostatic cysts. The majority of prostatic cysts are asymptomatic and originate in the posterior area of the prostate, such as in the Müllerian ducts and the utricle, as an embryological remnant; these cysts are observed in 0.5% to 7.9% of patients.^1^^,^^2^ However, these improved imaging techniques have increased the incidental determination of midline prostatic cysts (MPCs) in adult males, and the frequency of these findings is currently estimated to be 5-14%.^3^

Although the majority of the patients are symptom-free, enlarged prostatic cysts can compress adjacent structures, such as the posterior urethra, bladder neck or seminal vesicles, and then the patients may suffer obstructive or irritative voiding symptoms, recurrent urinary tract infections, epididymitis, chronic pelvic pain syndrome, hematospermia, low semen volume, or even infertility.^2^^,^^3^^,^^4^ Prostatic retention cysts rarely become symptomatic, but they may cause symptoms when the cyst enlarges to more than 3 cm. However, symptoms may occur even with smaller cysts if the location is just beside the bladder neck, and such cases are often misdiagnosed or confused with benign prostatic hyperplasia (BPH) or neuropathic bladder.^2^^,^^3^^,^^4^^,^^5^

We report on the case of a severely symptomatic, anteriorly located prostatic cyst arising from the bladder neck in a 30-year-old man who presented with lower urinary tract symptoms (LUTS), without any clinical evidence of benign prostatic hyperplasia or any endoscopic management. To our knowledge, symptomatic MPCs are generally located posteriorly and are rare. In fact, there are seven published reports of anteriorly positioned symptomatic MPCs arising from and obstructing the bladder neck with a ball-valve action during voiding.^6^^,^^7^^,^^8^^,^^9^^,^^10^^,^^11^^,^^12^

## CASE REPORT

A 30-year-old healthy man came to our outpatient clinic with a two-year history of severe LUTS, including frequent voiding, hesitancy, weak urinary stream and the sensation of residual urine. Despite alpha blocker drug treatment, his symptoms had worsened. His medical history was not significant in terms of previous urethral catheterization, urinary tract infection, pelvic/perineal trauma or neurological deficit. He had two children and did not have any ejaculatory complaints or infertility.

**Figure 1.**
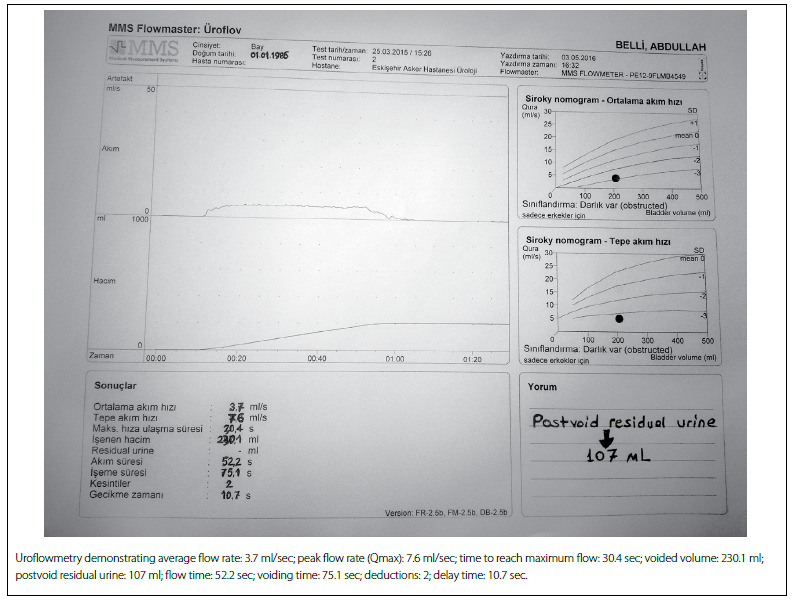
Preoperative uroflowmetry showing obstructed voiding.

**Figure 2.**
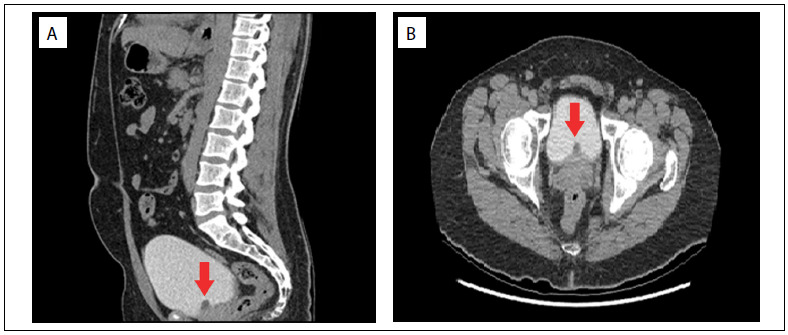
Sagittal (A) and axial (B) computed tomography urography images showing low-density small nodular lesion in the bladder neck that represents the anteriorly positioned midline prostatic cyst (arrow).

His International Prostate Symptom Score (IPSS) was 20, and his quality-of-life (QoL) score was 5. Digital rectal examination revealed a normal firm and nontender prostate without palpable nodules. A routine urine examination was normal and the culture was sterile. The urine cytology was not suggestive of malignancy, the serum prostatic specific antigen (PSA) level was 0.82 ng/ml (the reference value for PSA for the age range of 30-40 years is 0-2 ng/ml), and routine biochemical laboratory examinations were within normal limits. Uroflowmetry (Figure 1) demonstrated a peak flow rate (Qmax) of 7.6 ml/sec with a voided volume of 230 ml, and the postvoid residual urine volume was 107 ml (the reference values are voided volume > 150 ml and Qmax >15 ml/s). TRUS showed an anteriorly positioned prostatic cyst arising from the bladder neck and obstructing the bladder outlet. The cyst diameter was approximately 13 x 10 mm, inside a prostate with a volume of 22 ml. CT urography was performed to identify the origin of the cystic lesion and to rule out ectopic ureter or ureterocele (Figure 2).

**Figure 3.**
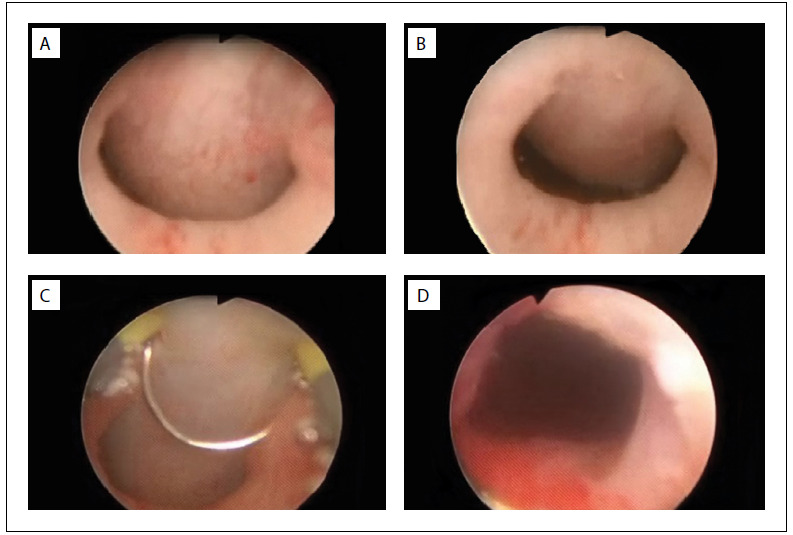
(A) and (B): anteriorly positioned midline prostatic cyst obstructing the bladder neck with action like a ball-valve; (C) and (D): transurethral unroofing of the cyst.

The patient then underwent cystourethroscopy examination under general anesthesia. This showed that the posterior wall of the prostatic urethra was normal and that there was cystic hemispherical bulging based on the anterior portion of the prostate. The bulge was located on the bladder neck at precisely twelve o’clock and was entirely compressing the bladder outlet. It was acting like a ball-valve, without lateral lobe hyperplasia (Figure 3). Moderate trabeculation due to gross back pressure change was also noted in the bladder. Transurethral marsupialization of the prostatic cyst to release the anatomical obstruction was performed, and milky fluid was expelled during the unroofing procedure.

A urethral catheter was left in place for two days, and the patient was then discharged. Histopathological examination revealed that the cyst wall was lined with benign flattened prostatic glandular epithelium without any preneoplastic change, which was consistent with the diagnosis of a prostatic retention cyst. At a return visit in the third postoperative month, a subjective dramatic improvement in symptoms was noted. IPSS was 5 and QoL was 2 at three months after the operation. Uroflowmetry showed an increased Qmax (18 ml/sec with a voided volume of 300 ml) and no residual urine. Furthermore, the patient had no symptoms suggestive of erectile dysfunction or ejaculation disorders.

## DISCUSSION

Although lower male genitourinary tract cystic lesions are uncommon and usually benign, they may be associated with a variety of genitourinary abnormalities and symptoms, such as urinary tract infection, chronic pelvic pain syndrome, postvoiding incontinence, recurrent epididymitis, prostatitis, obstructive and/or irritative voiding symptoms, hematospermia, low semen volume, ejaculatory pain, or even infertility.^2^^,^^3^^,^^4^^,^^9^^,^^13^

Galosi et al. reported that MPCs are seen by TRUS in 9.8% of cases.^1^ It has been reported that MPCs were observed in 7.6% of healthy men and 5% of symptomatic outpatients.^2^ Over recent years, new imaging techniques such as TRUS, CT and MRI have increased the incidental determination of MPCs in adult males, and their frequency is currently estimated to be 5-14%.^3^^,^^13^^,^^14^

However, the majority of prostatic cysts are asymptomatic. They can be categorized as symptomatic when the cyst’s presence is accompanied by infection or if its size and anatomical relationships affect the adjacent structures, which are most often located laterally.^14^ An analysis on 34 patients with symptomatic prostatic cysts by Tambo et al.^7^ revealed that 40% of the patients suffered from obstructive urinary symptoms, 33% from urinary retention, 9% from urodynia and 6% from infertility.

In 2009, Galosi et al.^1^ classified prostatic cysts into six distinct types based on TRUS and pathological correlation: isolated medial cysts, cysts of the ejaculatory duct, simple or multiple parenchymal cysts, complicated cysts (infectious or hemorrhagic), cystic tumors and secondary cysts relating to parasitic disease. MPCs are less common and are generally located posteriorly. They have traditionally been classified as Müllerian duct cysts and as enlarged prostatic utricles (mega-utricles), ejaculatory ducts, seminal vesicles and prostatic retention cysts.^15^^,^^16^ Furuya et al.^17^ classified MPCs, with or without the presence of sperm in the fluid content, using concomitant TRUS-guided opacification and dye injection. If there is communication with the seminal tract, then sperm can be found in the fluid content. They further classified MPCs into four categories: Type 1 MPC with no communication into the urethra (traditional prostatic utricle cyst); Type 2a MPC with no communication into the urethra [cystic dilatation of prostatic utricle (CDU)]; Type 2b CDU in communication with the seminal tract; and Type 3 cystic dilatation of the ejaculatory duct. They also found that the location, shape and volume of the MPC and the PSA level of the MPC fluid did not influence the classification.^18^ This finding may be useful for classifying various kinds of midline cysts of the prostate. However, in practice, this classification is not used because of the similarities of symptoms and primary treatment among prostate cysts.

According to the results from the pathological examination, our patient had a retention cyst of the prostate. This type of cyst is totally different from Müllerian duct cysts and prostatic utricle cysts, which are always lined with cuboidal or columnar epithelial cells. Retention cysts of the prostate gland are true acquired cysts and result from obstruction of prostatic glandular ductules, thus causing dilatation of the glandular acini. They can be located within any glandular zone of the prostate.^16^^,^^17^ Moreover, there are no sperm cells in the fluid obtained from prostatic retention cysts, and they usually do not cause symptoms. However, on rare occasions, they may cause obstructive symptoms if located close to the bladder neck, as in the case of our patient.^16^^,^^17^

Although approximately 35 patients with symptomatic prostatic cysts have been reported, there are only seven published reports on anteriorly located MPCs.^11^ Furthermore, to the best of our knowledge, only seven such cases of MPC of the bladder neck have been reported in the literature, as in our case. Also, this is the first case of an anteriorly located MPC of the bladder neck found in Turkey.^7^^,^^11^

Multiple therapeutic options have been described for management of symptomatic prostatic cysts, including transrectal aspiration with or without sclerotherapy, marsupialization with a transurethral technique and open surgery.^7^^,^^18^^,^^19^ Although recurrences of cysts that were incompletely excised during open surgery have been reported, recurrence-free results have been reported for medial prostatic cysts treated with the transurethral technique.^2^^,^^6^ Chang et al.^10^ previously reported successful results from transurethral resection of an MPC presenting with LUTS. Zhang et al.^20^ also recommended transurethral unroofing of small prostatic cysts (< 2 cm x 2 cm) that are close to the bladder cavity or urethra, because of the simplicity of the technique, the low risk of complications and the shorter convalescence period.

We searched for similar cases in different databases (PubMed, Embase and LILACS databases) using the terms: “prostatic cyst” AND “lower urinary tract symptoms” AND “midline” (Table 1). We found that few cases have been published.

**Table 1.**
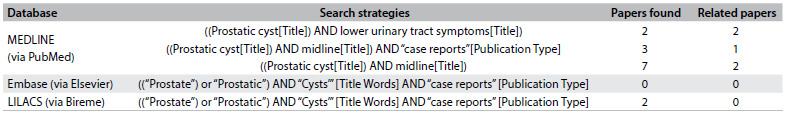
Search of the literature in medical databases for case reports on “Severe lower urinary tract symptoms due to anteriorly located midline prostatic cyst arising from the bladder neck in a young male”. The search was conducted on April 12, 2016

Our patient was suffering from obstructive voiding problems rather than irritative symptoms, and transurethral unroofing of the prostatic cyst, which was located anteriorly in the midline position, provided satisfactory results for his complaints. In this case, standard transurethral resection of the prostate was avoided in order to prevent antegrade ejaculation and erectile dysfunction, in the absence of the lateral lobe of prostatic hyperplasia.

## CONCLUSION

Although symptomatic anteriorly located MPCs of the bladder neck, as described in the case presented here, are uncommon, they should be borne in mind in the differential diagnosis of obstructive voiding symptoms, especially in young patients and patients who do not respond to medical therapy such as use of alpha-blockers. Transurethral unroofing of the cyst may provide safe treatment with successful and satisfactory results in selected cases.

## References

[1] Galosi AB, Montironi R, Fabiani A (2009). Cystic lesions of the prostate gland: an ultrasound classification with pathological correlation. J Urol.

[2] Dik P, Lock TM, Schrier BP, Zeijlemaker BY, Boon TA (1996). Transurethral marsupialization of a medial prostatic cyst in patients with prostatitis-like symptoms. J Urol.

[3] Furuya S, Hisasue S, Kato H, Shimamura S (2015). Novel insight for midline cyst formation in prostate: The involvement of decreased prenatal testosterone suggested by second-to-fourth digit ratio study. Int J Urol.

[4] Mayersak JS (1989). Urogenital sinus-ejaculatory duct cyst: a case report with a proposed clinical classification and review of the literature. J Urol.

[5] Pillai RG, Al Naieb ZA (2013). Successful Endoscopic Laser De-roofing of Simple Prostatic Cyst Causing Bladder Outlet Obstruction - A Case Study. Med Surg Urol.

[6] Nayyar R, Dogra PN (2009). Anteriorly placed midline intraprostatic cyst. J Endourol.

[7] Tambo M, Okegawa T, Nutahara K, Higashihara E (2007). Prostatic cyst arising around the bladder neck-cause of bladder outlet obstruction: two case reports. Hinyokika Kiyo.

[8] Issa MM, Kalish J, Petros JA (1999). Clinical features and management of anterior intraurethral prostatic cyst. Urology.

[9] Yildirim I, Kibar Y, Sümer F (2003). Intraurethral prostatic cyst: a rare cause of infravesical obstruction. Int Urol Nephrol.

[10] Chang SG, Hwang IC, Lee JH, Park YK, Lim JW (2003). Infravesical obstruction due to benign intraurethral prostatic cyst. J Korean Med Sci.

[11] Lee JY, Kang DH, Park HY (2010). An anteriorly positioned midline prostatic cyst resulting in lower urinary tract symptoms. Int Neurourol J.

[12] Diaz RR, Lee JY, Choi YD, Cho KS (2013). Unroofed midline prostate cyst misled into a stricture with obliterative bladder neck contracture following a laser prostatectomy. Int Neurourol J.

[13] Ishikawa M, Okabe H, Oya T (2003). Midline prostatic cysts in healthy men: incidence and transabdominal sonographic findings. AJR Am J Roentgenol.

[14] Nayyar R, Wadhwa P, Dogra PN (2008). Midline intraprostatic cyst: An unusual cause of lower urinary tract symptoms. Indian J Urol.

[15] Juárez Soto A, Ribè Subirà N, Manasia P (2004). Classification of cystic structures located at the midline of the prostate: our experience. Arch Ital Urol Androl.

[16] Nghiem HT, Kellman GM, Sandberg SA, Craig BM (1990). Cystic lesions of the prostate. Radiographics.

[17] Furuya R, Furuya S, Kato H (2008). New classification of midline cysts of the prostate in adults via a transrectal ultrasonography-guided opacification and dye-injection study. BJU Int.

[18] Stricker HJ, Kunin JR, Faerber GJ (1993). Congenital prostatic cyst causing ejaculatory duct obstruction: management by transrectal cyst aspiration. J Urol.

[19] Saito S (2002). Transrectal ultrasound-guided puncture, drainage, and minocycline hydrochloride sclerotherapy for the symptomatic prostatic cyst. J Endourol.

[20] Zhang H, Qi F, Wang J, Chen M, Li Z, Zu X (2011). Midline Prostatic Cysts Presenting with Chronic Prostatitis or Secondary Infertility and Minimally Invasive Treatment: Endoscopic or Laparoscopic Approach?. Surgical Science.

